# Utilization of the Maryland Environmental Justice Screening Tool: A Bladensburg, Maryland Case Study

**DOI:** 10.3390/ijerph16030348

**Published:** 2019-01-26

**Authors:** Aubree Driver, Crystal Mehdizadeh, Samuel Bara-Garcia, Coline Bodenreider, Jessica Lewis, Sacoby Wilson

**Affiliations:** 1 Public Health Science Program, University of Maryland, 255 Campus Drive, College Park, MD 20740, USA; adriver@umd.edu (A.D.); sbaragarcia13@gmail.com (S.B.-G.); 2 Environmental Science and Technology Department, University of Maryland, 1451 Animal Science Bldg, College Park, MD 20742-2315, USA; cbodenr1@umd.edu; 3 Department of Psychology, Swarthmore College, 500 College Ave, Swarthmore, PA 19081, USA; Jlewis2@swarthmore.edu; 4 Maryland Institute for Applied Environmental Health, University of Maryland, 255 Valley Drive, College Park, MD 20742, USA; swilson2@umd.edu

**Keywords:** EJSCREEN, geographic information systems, environmental justice, CalEnviroScreen, vulnerable populations

## Abstract

Maryland residents’ knowledge of environmental hazards and their health effects is limited, partly due to the absence of tools to map and visualize distribution of risk factors across sociodemographic groups. This study discusses the development of the Maryland EJSCREEN (MD EJSCREEN) tool by the National Center for Smart Growth in partnership with faculty at the University of Maryland School of Public Health. The tool assesses environmental justice risks similarly to the U.S. Environmental Protection Agency’s (USEPA) EJSCREEN tool and California’s tool, CalEnviroScreen 3.0. We discuss the architecture and functionality of the tool, indicators of importance, and how it compares to USEPA’s EJSCREEN and CalEnviroScreen. We demonstrate the use of MD EJSCREEN through a case study on Bladensburg, Maryland, a town in Prince George’s County (PG) with several environmental justice concerns including air pollution from traffic and a concrete plant. Comparison reveals that environmental and demographic indicators in MD EJSCREEN most closely resemble those in EPA EJSCREEN, while the scoring is most similar to CalEnviroScreen. Case study results show that Bladensburg has a Prince George’s environmental justice score of 0.99, and that National Air Toxics Assessment (NATA) air toxics cancer risk is concentrated in communities of color.

## 1. Introduction

Environmental justice research since the 1970s has illustrated siting disparities of environmental hazards and locally unwanted land uses (LULUs) in communities of color [1,2,3,4,5]. Specifically, these neighborhoods are disproportionately burdened by noxious facilities that threaten the social, environmental, and physical health of local residents [5,6,7,8,9]. There are a number of environmental stressors that affect the physical and social well-being of a population such as: (1) proximity to hazardous waste sites, (2) exposure to air and water pollution, (3) residential crowding, (4) high levels of ambient noise, (5) the work environment, and (6) quality of local schools [8,9,10,11]. These harmful exposures have been examined using Geographic Information System (GIS) and Public Participatory Geographic Information System (PPGIS) mapping tools. The use of GIS and PPGIS in public health research has produced a wealth of information that can be used to improve the quality of life in overburdened neighborhoods.

GIS is a mapping tool which can be used to visualize the spatial distribution of pathogenic and salutogenic elements in the environment [12]. GIS is central to studying environmental justice because it illustrates how social, economic, and racial stratification have reinforced the disproportionate burden of environmental health hazards on marginalized communities. Studies using this technology have found that low income and communities of color are more likely to be located near pollution sources [13,14,15,16]. GIS has also been utilized in studies of traffic-related air pollution (TRAP) and water pollution in overburdened communities [17,18,19,20]. The presence of such environmental hazards, as well as walkable streets and parks, food quality around schools, and supermarket accessibility have all served as indicators of community wellness [21,22,23].

These findings have been mirrored by PPGIS studies on environmental hazards in vulnerable communities [13,24]. Studies utilizing this visualization approach have stressed the importance of incorporating the lived experiences of community members in environmental health research [25,26,27]. PPGIS integrates local and government knowledge on pollution sources to build community capacity and empower citizens to take active roles in environmental justice discussions [27,28,29]. PPGIS has been used to investigate the presence of urban blue spaces and aquatic environments, urban green spaces, community revitalization efforts, national forests, and land use [26,30,31,32,33,34].

GIS and PPGIS serve as crucial components of the California Communities Environmental Health Screening tool (i.e., CalEnviroScreen), which was created by the Office of Environmental Health Hazard Assessment (OEHHA) and California Environmental Protection Agency (CalEPA) in 2013. CalEnviroScreen uses mapping and screening techniques at the census-tract level to identify vulnerable communities that are disproportionately burdened by environmental hazards in the state of California [10]. The tool assigns cumulative impact scores, also referred to as CalEnviroScreen Scores, to each census tract as a measure of environmental inequality [8,35].

Another tool which applies a similar methodology on a national level is known as EJSCREEN (hereby referred to as EPA EJSCREEN for clarity), released in 2015 by the United States Environmental Protection Agency (US EPA). The tool’s purpose is to consolidate demographic and environmental health data from a multitude of publicly available sources into maps and reports [36,37]. This enables researchers and the general public to compare the state of environmental justice in marginalized communities to state, regional, and national averages [36,37]. 

CalEnviroScreen and EPA EJSCREEN exist to examine the environmental burdens within an area of interest. A team from the National Center for Smart Growth and School of Public Health helped to develop a preliminary version of a screening tool for the state of Maryland known as MD EJSCREEN. MD EJSCREEN was built upon the framework of the aforementioned tools using feedback gathered from stakeholders and community members in Prince George’s County. In attempts to further develop another mapping tool, MD EJSCREEN was created to focus on identifying disadvantaged communities. Although EPA EJSCREEN is able to map the entire US, it is unable to address all issues relevant at the local level due to the broad scope of the tool. MD EJSCREEN incorporates additional indicators that are more specific to Maryland such as: asthma emergency discharges and watershed failure. In addition, EPA EJSCREEN differs in its scale of analysis, and its ability to compute a score of environmental justice like MD EJSCREEN and CalEnviroScreen, making the development of a tool specific to Maryland all the more necessary.

This paper will discuss the development of MD EJSCREEN, community feedback received during a series of demonstration workshops, the architecture of the tool, how it compares to other environmental justice visualization tools, scoring methodology, and its application for Bladensburg, Maryland, a town in Prince George’s County.

## 2. Materials and Methods 

Through a series of demonstration workshops, feedback was gathered from groups of stakeholders including Prince George’s County residents in order to develop, improve, and determine any and all environmental, population, and health indicators that would be included in MD EJSCREEN. The majority of the stakeholders included Prince George’s County Environmental Action Council (EAC) members, Port Towns residents, the environmental justice legislative team (EJLT), and the statewide Commission on Environmental Justice and Sustainable Communities. In order to obtain feedback on the indicators that were deemed necessary and acceptable for MD EJSCREEN, posters containing lists of different indicators were displayed at the stakeholder meetings. Surveys were also distributed to all stakeholders to gather feedback regarding the decidedly necessary and acceptable indicators that were to be highlighted in MD EJSCREEN.

The first stakeholder meeting occurred with the Prince George’s County EAC, an organization that presents feedback on environmental concerns to the Prince George’s County Department of the Environment. The feedback concerning each indicator was documented on flashcards and shared with the EJSCREEN development team. This information was used to build MD EJSCREEN.

Posters highlighting the relevance of each specified indicator were displayed at other stakeholder meetings. For instance, posters were displayed on site at Bladensburg Waterfront Park, and attendees were instructed to rank the importance of each indicator in the community. Four stickers were used to capture feedback from the stakeholders on listed indicators: (1) green, (2) blue, (3) yellow, and (4) red symbolizing very important, important, somewhat important, and not important, respectively. Unfortunately, the significance of each colored sticker became inconsistent for each individual stakeholder. To account for this issue, we removed the important/not important value from each sticker, and instead counted the physical number of stickers placed on each indicator to properly reflect the significance of each indicator. In the development of MD EJSCREEN, community members advocated for the inclusion of seven indicators to highlight the aspects of economic, environmental, and exposure factors that can significantly alter community health. The seven indicators were: (1) asthma emergency room discharges; (2) percent non-White; (3) proximity to treatment, storage, and disposal facilities (TSDFs); (4) myocardial infarction discharges; (5) low birth weight infants; (6) particulate matter (PM_2.5_); and (7) pathogenic infrastructure. Of these indicators, only pathogenic infrastructure was not mapped due to lack of sufficient data. 

Additional feedback was obtained through surveys that asked community members to prioritize indicators taken from EPA EJSCREEN, CalEnviroScreen, and new indicators suggested by community members. A Likert scale was used to rank indicators by importance, in which a score of 1 indicated that said indicator was of low priority and a score of 5 indicated that it was of high priority. These surveys were distributed to several stakeholder groups including, the Association of Baltimore Area Grantmakers (ABAG), the Commission on Environmental Justice and Sustainable Communities, the Prince George’s County EAC, and the EJLT.

## 3. Results

**Figure 1:**
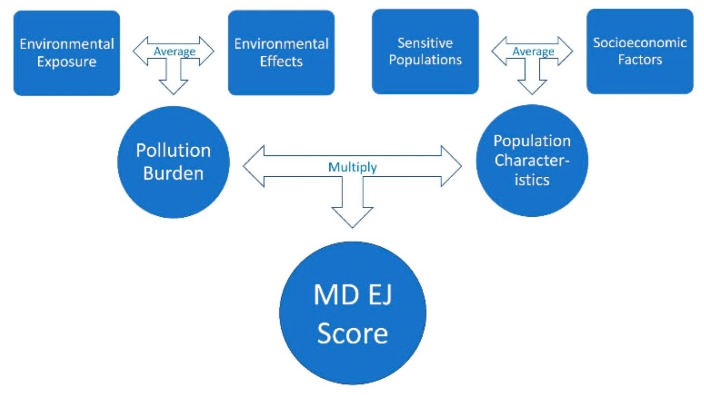
Overview of MD EJ (Maryland Environmental Justice) Scoring Process. The Environmental Exposure and the Environmental Effects Scores are averaged to calculate the Pollution Burden, and the Sensitive Populations and Socioeconomic Factors are averaged to create the Population Characteristics Score. The Pollution Burden and Population Characteristics are then multiplied to calculate the final MD EJ Score.

MD EJSCREEN allows users to create interactive maps of demographic and environmental health within four primary categories: (1) Pollution Burden: Exposure; (2) Pollution Burden: Effect; (3) Population Characteristics: Sensitive Populations; and (4) Population Characteristics: Socioeconomic Factors. Each of these categories (Figure 1) contain a number of environmental justice indicators which are defined in Table 1.

### 3.1. Scoring Process

MD EJSCREEN illustrates the extent of environmental injustice in an area by assigning an EJ Score to each census tract. To calculate the MD EJ score, the tool applies the methodology used by CalEnviroScreen [8]. A MD EJ score is calculated by first converting the raw indicator scores within each of the four categories: (1) Pollution Burden: Exposure; (2) Pollution Burden: Effect; (3) Population Characteristics: Sensitive Populations; and (4) Population Characteristics: Socioeconomic Factors into percentiles. The raw indicator scores are ranked from lowest to highest at the census-tract level, and then assigned percentile values from 0 to 1 based on how the indicator scores compare to other census tracts in the state. MD EJSCREEN also produces a PG EJ score, which applies the same methodology, but assigns percentile values from 0 to 1 based on how the indicator scores compare to other census tracts in Prince George’s County. 

These values are averaged to create two scores per census tract: (1) the Population Characteristics score, and (2) the Pollution Burden score. The Population Characteristics score is the average of all indicators in the Sensitive Populations and Socioeconomic Factors categories. The Pollution Burden score is the average of all indicators in the Environmental Effects and Exposures categories. In this calculation; however, the Environmental Effects component is half-weighted. This means that the Environmental Effects category has a weight equal to 1/3, while the Environmental Exposure category has a weight equal to 2/3. This is done because Environmental Effects make a smaller contribution to Pollution Burden than Environmental Exposures. 

To calculate the EJ scores, the Population Characteristics score is multiplied by the Pollution Burden score [38]. This score is reported as a percentile value 0 to 1 based on how it compares to other census tracts. An EJ score illustrates the magnitude of environmental justice concern in an area; thus, areas with high concentrations of low-income and/or non-White populations and high exposures to environmental hazards will have scores closer to 1. 

### 3.2. Comparison of MD EJSCREEN to CalEnviroScreen and EPA EJSCREEN

While MD EJCREEN, CalEnviroScreen, and EPA EJSCREEN all aid users in increasing environmental awareness, there are a number of differences in the usability, functionality, and type of information used in each tool. For instance, EPA EJSCREEN contributes useful demographic and environmental indicator data; however, the broad national scope of the tool limits the specificity and relevance of a given block group or census tract. CalEnviroScreen on the other hand, provides specific population characteristics and pollution burden data for the state for California and more importantly, has proved to be an apt model for MD EJSCREEN. However, CalEnviroScreen is missing pertinent information on Maryland specific indicators such as lead paint prevalence.

NATA respiratory hazard index, and Proximity to risk management plan (RMP) sites. A comparison of the usability and functionality of each tool is provided in Table 3.

In terms of specific indicators, the tools have a number of similarities and differences. Table 1 displays a comparison of indicators included in CalEnviroScreen, EPA EJSCREEN, and MD EJSCREEN. All three tools include: (1) NATA DPM, (2) PM_2.5_, (3) ozone, (4) traffic proximity and volume, (5) proximity to national priority list (NPL) sites, (6) percent non-White, (7) percent low-income, (8) less than high school education, and (9) linguistic isolation as indicators. MD EJSCREEN and EPA EJSCREEN share seven indicators: (1) NATA air toxics cancer risk, (2) NATA respiratory hazard index, (3) lead paint indicator, (4) proximity to RMP sites, (5) proximity to TSDFs, (6) individuals under age 5, and (7) individuals over age 64. CalEnviroScreen and MD EJSCREEN share three indicators: (1) myocardial infarction discharged, (2) low birth weight infants, and (3) unemployment. CalEnviroScreen includes eight unique indicators, by far the highest amount, while MD EJSCREEN has two, and EPA EJSCREEN has none.

In the development of MD EJSCREEN, feedback was gathered on what specific indicators stakeholders felt should be included in the tool, including indicators that highlight the aspects of economic, environmental, and exposure factors that can significantly alter community health. Table 2 includes additional indicators that were advocated for by community members, some of which are already in the ‘Additional Context Layers’ category in MD EJSCREEN. Once sufficient data is obtained for the indicators not already included in the tool, they will be added to this category.

In addition to the differences in indicators mapped by the three tools, shown in Table 1 above, CalEnviroScreen, EPA EJSCREEN and MD EJSCREEN also differ in their usability and functionality as shown in Table 3. In our analysis of usability and functionality of the three tools, we identified the main features of GIS tools including searching locations, navigation (zoom in/out), printing maps, sharing maps, and creating maps [64]. We also compared additional features that we identified by searching through the tools. We then classified these features as assets of usability or functionality. We defined usability as, “the extent to which a product can be used by specified users to achieve specific goals with effectiveness, efficiency and satisfaction in a specified context of use” [65]. We expand on this definition by referring to aspects of the tool that can be controlled by the user as ‘usability’. 

All three tools have user-defined search, user-defined location markers, and zoom in/out features. Only EPA EJSCREEN has user-defined base map options, and the ability to bookmark maps in the tool for future reference. Both EPA EJSCREEN and CalEnviroScreen have additional resources such as help manuals or explanatory videos that users may reference. Currently, MD EJSCREEN does not possess these features. A training guide and videos will be released once MD EJSCREEN is in its final stages of development.

Functionality is defined as the range of operations that can be performed by the tool. Currently, the features all three tools have in common are their ability to generate coordinates for a user-defined location and to create maps. EPA EJSCREEN can map a number of environmental and demographic indicators, and can generate side-by-side maps in place of overlaying different indicators on the same map. The tool can also map demographic data from the 2012 to 2016 ACS, and the 2000 and 2010 censuses. Additionally, EPA EJSCREEN gives users the option to search for already made maps using GeoPlatform. It also possesses a broad range of other functions such as the option to print custom maps, create custom reports and graphs, measure distances, and download raw data. Moreover, it has a mobile version of the tool that users can access on smartphones and tablets.

CalEnviroScreen allows users to map each individual indicator or CalEnviroScore in an individual window, with no option to overlay maps or make side by side maps. It also provides users with the options to print maps and download raw data, but also includes a ‘share’ feature that allows users to share information over social media, statistics, and create scores. While CalEPA does produce a general report for the state of California with the 2018 update of CalEnviroScreen, the maps and data presented in the report cannot be customized by the user [35]. As expected for a provisional tool, MD EJSCREEN has a relatively limited range of capabilities, and has no print or share feature. However, it is able to overlay indicators with one another, allowing for easy visual comparison. It also displays legislative districts and county lines, allowing users to easily identify state and local governments to which they can address concerns.

### 3.3. Bladensburg: A Case Study

**Figure 2:**
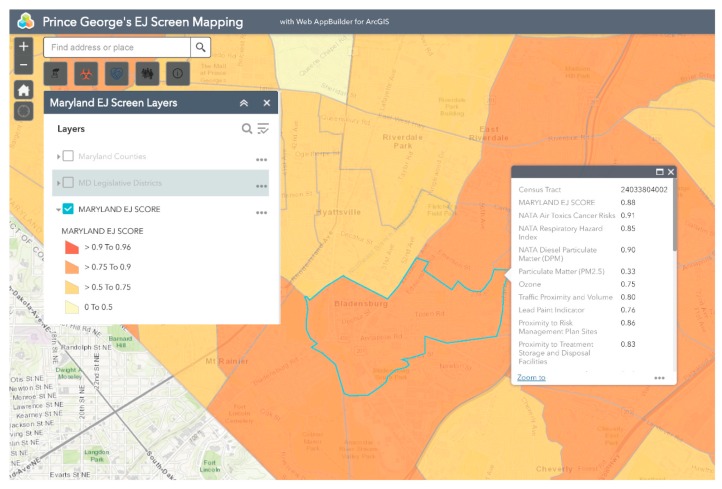
MD EJ Score for Bladensburg. This screenshot from the MD EJSCREEN mapping tool shows the MD EJ Score layer for Bladensburg, MD.

**Figure 3:**
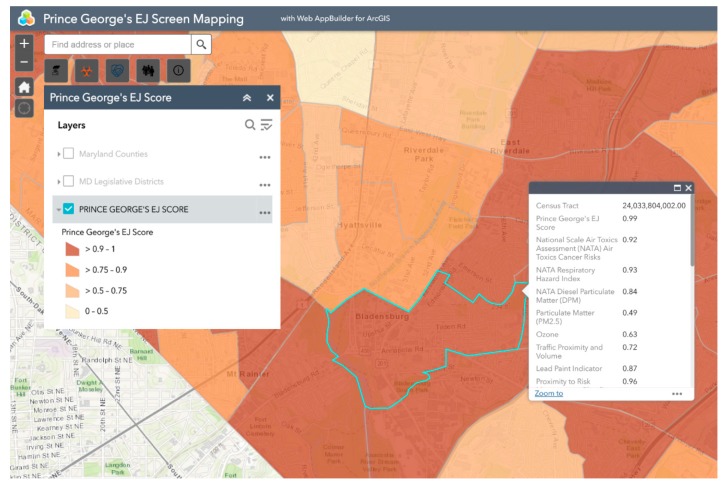
PG EJ score for Bladensburg. This screenshot from the MD EJSCREEN mapping tool shows the PG EJ score of Bladensburg, MD.

**Figure 4:**
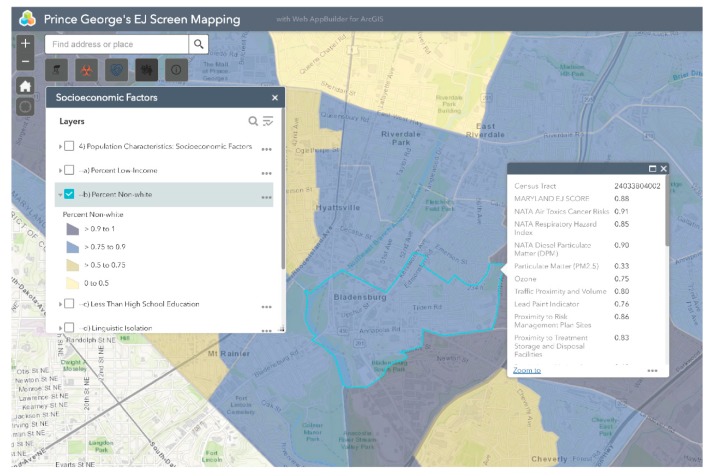
Percent non-White in Bladensburg. This map highlights the rate of percent non-White individuals in Bladensburg, MD.

**Figure 5:**
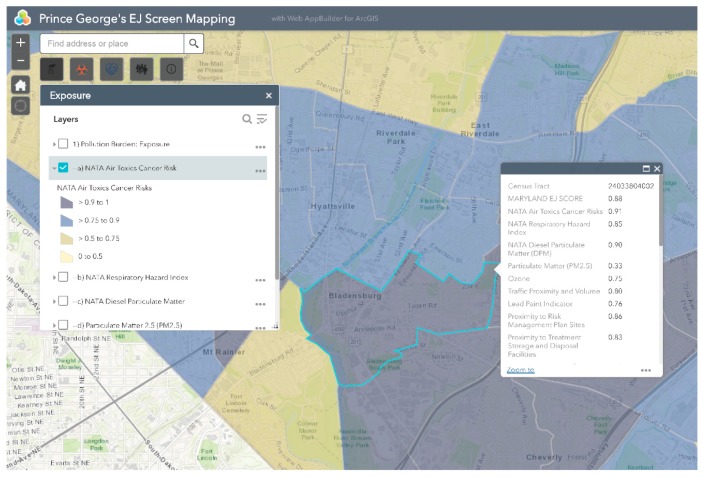
NATA air toxics cancer risk in Bladensburg. This screenshot from the MD EJSCREEN mapping tool shows the percentile for the NATA air toxics cancer risk in Bladensburg, MD.

**Figure 6:**
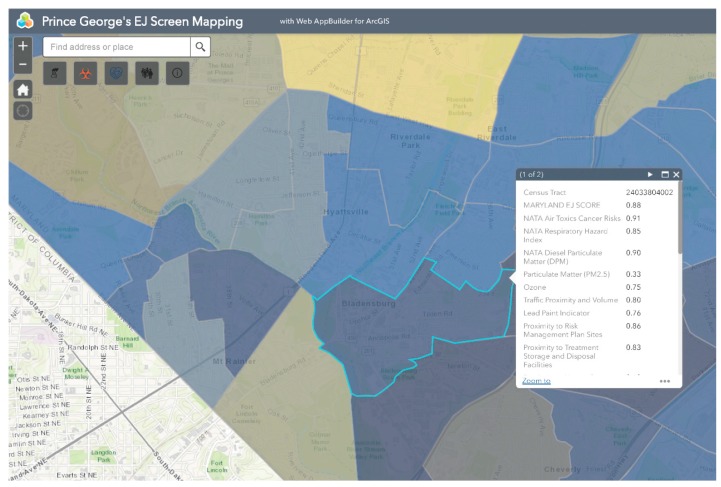
NATA air toxics layer overlaid with percent non-White in Bladensburg.

**Figure 7:**
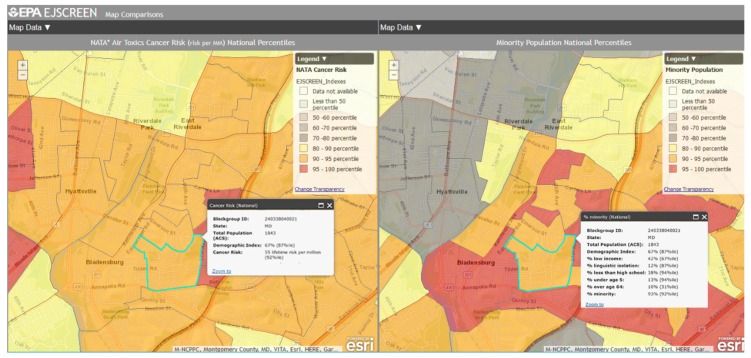
EPA EJSCREEN side-by-side maps displaying national percentiles for NATA air toxics cancer risk (**left**) and non-White population (**right**).

Bladensburg is a town in Prince George’s County that has experienced environmental injustice due to racial and economic stratification. The Bladensburg population is primarily Black (62.7%) and Latinx (33.0%), with small concentrations of White (13.9%) and Asian (1.6%) residents [66]. Studies have shown that proximity to hazards is directly related to race/ethnicity, with one study specific to Maryland, Louisiana, and West Virginia finding that African-Americans are more likely to be located in close proximity to Toxic Release Inventory (TRI) facilities [67,68,69,70,71]. In comparison to the national average of 14%, 20.1% of Bladensburg residents live below the federal poverty line [66]. Due to underlying social and economic vulnerabilities, residents in Bladensburg are more likely to experience low property values and the disproportionate siting of environmental hazards in their communities than their more affluent counterparts [72,73]. Bladensburg residents also endure a large pollution burden, with heavy commuter and industrial traffic and a concrete block plant acting as sources of particulate matter (PM), volatile organic compounds (VOCs), and polycyclic aromatic hydrocarbons (PAHs) [74]. Due to their exposure to air pollutants, Bladensburg residents are at increased risk for respiratory problems and cancer [72,75,76,77]. An example analysis of environmental injustice in Bladensburg using MD EJSCREEN is presented below (Figure 2, Figure 3, Figure 4, Figure 5 and Figure 6). For the purpose of the Bladensburg case study, the PG EJ score, a separate score created by the tool, is used, in addition to the MD EJ score (Figure 2). The PG EJ score compares the Bladensburg census tract to the rest the census tracts in Prince George’s County, while the MD EJ score compares the Bladensburg census tract to all census tracts in Maryland. A comparison with EPA EJSCREEN is provided for Bladensburg in Figure 7. 

For example, this map illustrates the PG EJ score for Bladensburg. The PG EJ score is represented as a percentile, meaning that Bladensburg has an EJ score higher than 99% of the census tracts in Prince George’s County. Furthermore, the score is an overall indication of the prevalence of environmental hazards in the Bladensburg area.

This map visualizes the percent non-White population in Bladensburg. Bladensburg appears within the 0.75–0.90 percentile range, meaning that its percent non-White population is higher than 75–90% of the census tracts in the state or county.

This map visualizes NATA air toxics cancer risk in Bladensburg. Bladensburg appears within the 0.9–1 percentile range, meaning that its NATA air toxics cancer risk is higher than 90–100% of the census tracts in the state or county.

This map visualizes the percent non-White population in Bladensburg in relation to NATA air toxics cancer risk. Bladensburg appears within the 0.9–1.0 percentile range, meaning that the calculated risk of developing cancer due to air pollution is higher than the risk in 90–100% of the census tracts in the state or county. Coupled with the high percentage non-White population, these findings illustrate the disproportionate exposure to human and environmental health hazards experienced by populations of color.

While MD EJSCREEN conducts analyses at a larger scale by organizing data by census tract, EPA EJSCREEN analyzes data on a smaller scale, by block group. According to Figure 7, this block group is 97% non-White and it is in the 95th percentile. This means that the percent non-White in this block group is greater than or equal to the percent non-White where 95% of the US population lives. The lifetime NATA cancer risk for this block group is 55 people per 1 million. This area is also in the 92nd percentile, meaning that the lifetime NATA cancer risk in this block group is greater than or equal to the risk where 92% of the US population lives. 

## 4. Discussion

Despite the presence of tools such as EPA EJSCREEN and CalEnviroScreen, stakeholders expressed the need for a mapping tool which caters to specific environmental health concerns in Maryland. Other EJ mapping tools lack pertinent information on indicators specific to Maryland communities such as lead paint prevalence, NATA respiratory hazard index, and proximity to RMP sites. EPA EJSCREEN can map useful demographic and environmental indicators, but is hindered by its broad scope, and the use of block groups as the geographic unit of analysis. 

EPA EJSCREEN was not developed with the intention of determining the presence or absence of environmental injustice in communities. All conclusions drawn from the tool must be substantiated with additional information at the local level in order to perform a true assessment of environmental justice. Due to the broad scope of the tool, it is unable to address all issues relevant at the local level. Therefore, many environmental concerns central to communities are not shown.

EPA EJSCREEN also identifies marginal uncertainty in EJ Index values when mapping block groups. Mapping an individual block group can identify pollution ‘hot spots’, but produces uncertainty because the tool must estimate the location of residences. Due to such uncertainties, the tool cannot confidently compare or rank indicators when only minor differences exist between percentile scores. In order to avoid such uncertainties, the US EPA recommends that users create a buffer by applying the tool to a larger geographic area that covers multiple block groups, but which may overlook ‘hot spots’ [36]. We seek to address this weakness in MD EJSCREEN by conducting analysis at a larger scale (using a census tract), rather than by block group. 

As mentioned previously, only CalEnviroScreen and MD EJSCREEN are able to produce EJ scores for a user-specified census tract. The computation of an EJ score allows for clear and succinct analysis of the level of environmental injustice in an area of concern. EPA EJSCREEN cannot produce cumulative EJ scores but does produce EJ indices. An EJ index combines demographic indicators with a single environmental indicator, resulting in 11 EJ indexes that reflect the 11 environmental indicators in the tool. EJ indexes apply the idea of ‘excess risk’ by examining the disparities between block group averages and nation averages for environmental indicators across different demographic indicators. EJ indices can therefore be used to identify geographic locations which may burdened by environmental hazards. Despite this, EJ indices are limited in their applications to environmental justice because they do not consider the cumulative impacts of multiple environmental stressors on the health and well-being of a population. 

Research has repeatedly shown that the concentration of environmental hazards elevates stress and other adverse health outcomes, while simultaneously decreasing quality of life and community sustainability [8,78,79,80]. Environmental stressors include water contamination, hazardous waste, land used for incinerators and landfills, and lack of green space [78]. MD EJSCREEN provides a more accessible and interactive way for residents to grasp how their health is affected by the built environment, visualize trends in who is directly affected and to what degree, and create long-lasting change in their community.

The US EPA has set environmental health standards, yet, these programs have not specifically confronted the issue of cumulative impacts on non-White populations [81]. This makes mapping tools such as EJSCREEN, CalEnviroScreen, and MD EJSCREEN significant in determining how populations of color suffer an unjust burden of environmental risks. The scores available in MD EJSCREEN allow residents and other stakeholders to compare the rates of pollution, the effects of pollution, and the most vulnerable subpopulations to other areas of the state. For instance, users can easily visualize that areas with a higher percentage of low-income residents also tend to be located near areas with high traffic volume and PM_2.5_, as well as the potential health risks associated with those locations [81]. These scores can be used by residents to better advocate for their health, and government officials to determine specific changes that need be made to reduce environmental injustice. 

The ability of MD EJSCREEN to identify areas of environmental justice concern has been mirrored by other tools such as CalEnviroScreen. Researchers utilizing this technology found that non-White individuals are more likely to live near pollution sources than non-Hispanic Whites, and that they also experience higher concentrations of poverty, ozone, DPM, pesticide use, solid waste sites, and gas-fired power plants [8,82]. Additional studies isolating the Latinx population found that they are especially vulnerable to environmental injustice due factors such as linguistic isolation, racial segregation, systematic housing market discrimination, real estate steering practices, and blocked channels of residential and economic mobility [83,84]. Additionally, socioeconomic indicators such as education and income were found to be strongly associated with levels of disease burden [10,85].

CalEnviroScreen has also been used to link vulnerable populations to visionary policies capable of transforming overburdened areas into healthy, thriving communities. For example, California Environmental Justice Alliance (CEJA)’s Green Zones project applies the CalEnviroScreen tool to identify concentrations of industrial pollution sources thereby streamlining resources, regulatory attention, and sustainable economic development to vulnerable communities [86]. The term ‘Green Zone’, refers to mostly low-income communities of color that have organized against discriminatory land use patterns and for neighborhood restoration that addresses economic, social, and environmental health concerns [86].

The EPA’s Clean Power Plan employed EPA EJSCREEN to inform policy that addresses the disproportionate siting of environmental hazards in communities of color, low income, and indigenous communities by performing proximity-based analyses of power plants, taking into account demographics and cumulative impacts [87]. The analyses revealed that in comparison to nation averages, low income and people of color are more likely to live near power plants [83]. The proximity analysis outlined by the Clean Power Plan allows states to improve access to energy efficiency (EE), renewable energy (RE), and financial assistance programs [87].

Similarly, the North Carolina Department of Environmental Quality (NC DEQ) conducted an environmental justice assessment using EPA EJSCREEN for a proposed fly ash project at the Duke Energy Lee plant in Goldsboro, North Carolina. The EPA EJSCREEN analysis revealed that the per capita income of individuals living within two miles of the Lee plant is $17,847 per year, less than the Goldsboro average of $19,243, and the state average of $26,779. This information demonstrates that if the plant is to expand, the pollution burden would fall primarily on low-income communities [88].

EPA EJSCREEN has also been used to address the environmental health concerns of incarcerated populations. Currently, the tool is at the forefront of the ‘prison ecology movement’, which utilizes GIS mapping to examine the proximity of correctional facilities to known hazardous waste sites [89]. Studies performed by the Prison Ecology Project (PEP) found that among a range of federal and state prisons from Colorado to Indiana, 589 were located within three miles of a Superfund or hazardous waste site [90].

In conducting this study, we encountered multiple barriers to obtaining reliable feedback on the indicators. The amount of information we received for each indicator was not as complete as it could have been due to low response rates from stakeholders. Other limitations include the accuracy of the feedback received during the stakeholder meeting held in Bladensburg, Maryland. Due to these limitations, the utility of the additional indicators included within the ‘Additional Context’ category to the Prince George’s community, may be lower than anticipated. Nevertheless, the findings of this study can be further implemented in various future EJ investigations. 

Presently, we are working to increase the functionality and usability of MD EJSCREEN so it has similar capabilities to EPA EJSCREEN and CalEnviroScreen particularly geostatistical analysis. In the future, we will add the ability of users to upload their own qualitative or quantitative data collected via citizen science or another scientific approach. Additionally, MD EJSCREEN will be expanded to the entire state of Maryland, enabling more communities to engage with and take control of environmental justice in their neighborhoods. As the tool expands, more pertinent indicators specific to Maryland could be incorporated based on feedback from stakeholders across the state, strengthening the tool and its ability to combat environmental injustice. Although no stakeholder evaluations have been conducted yet, once the tool is in its final stage of development, we will perform an evaluation of stakeholder satisfaction with the tool. These evaluations will focus on the major metrics used to assess the functionality and usability of GIS mapping tools. This will allow us to understand if the tool has achieved its primary objective: to represent the environmental justice concerns specific to Maryland residents.

## 5. Conclusions

MD EJSCREEN can be used to make public health improvements for all communities, allowing residents to advocate for new policies and better enforcement of policies. In addition, government officials can use the information provided by this tool to identify pressing concerns of their constituents and implement more equitable policies. A comprehensive training guide and video showing how to navigate and use the tool will be developed and released for community members and stakeholders. A series of training sessions will serve to provide technical assistance on an as-needed basis, making it easier to apply MD EJSCREEN in policy decisions and community advocacy.

## Figures and Tables

**Table 1:** Comparison of EJ (Environmental Justice) screening tool indicators.

Indicators	Description	EPA EJSCREEN	CalEnviroScreen	MD EJSCREEN
**Pollution Burden: Exposure**				
National Scale Air Toxics Air (NATA) Toxics Cancer Risk	Lifetime risk of developing cancer from inhalation of air toxins. Reported as risk per lifetime per million people [36].	X		X
NATA Respiratory Hazard Index	Air toxics respiratory hazard index. This is the sum of hazard indices for those air toxics with reference concentrations based on respiratory endpoints, where each hazard index is the ratio of exposure concentration in the air to the health-based reference [36].	X		X
NATA Diesel Particulate Matter (DPM)	Levels of diesel particulate matter in air. Reported as micrograms per cubic meter (µg/m^3^) [35,36].	X	X	X
Particulate Matter (PM_2.5_)	Levels of particulate matter with a diameter of 2.5 micrometers or smaller in air. Reported as micrograms per cubic meter (µg/m^3^) [35,36].	X	X	X
Ozone	Summer seasonal average of the maximum daily 8-hour concentration of ozone in air in parts per billion [35,36].	X	X	X
Traffic Proximity and Volume	Count of vehicles (average annual daily traffic) at major roads within 500 meters or close to 500 meters, divided by distance in meters [35,36].	X	X	X
Pesticide Use	Total pounds of selected active pesticide ingredients (filtered for hazard and volatility) used in production-agriculture per square mile, averaged over three years (2012 to 2014) [36].		X	
Drinking Water Contaminants	Water tested to contain one or more contaminants listed in ‘Update to California Communities Environmental Health Screening Tool’. Reported as yearly averages of chemical contaminant concentrations for each census tract [36].		X	
Toxic Releases from Facilities	Toxicity-weighted concentrations of modeled chemical releases to air from facility emissions and off-site incineration (averaged over 2011 to 2013) [36].		X	
**Pollution Burden: Environmental Effects**				
Lead Paint Indicator	Percent of houses built before 1960, which likely contain lead paint [36].	X		X
Proximity to Risk Management Plan (RMP) Sites	Count of RMP (potential chemical accident management plans) facilities within 5 kilometers or close to 5 kilometers, divided by distance in kilometers [36].	X		X
Proximity to Treatment Storage and Disposal Facilities (TSDF)	Count of TSDF (hazardous waste management facilities) within 5 kilometers or closest to 5 kilometers, divided by distance in kilometers [36].	X		X
Proximity to National Priorities List (NPL) Sites	Count of NPL/Superfund sites (polluted sites that pose a risk to human health and/or the environment) within 5 kilometers or close to 5 kilometers, divided by distance in kilometers [35,36].	X	X	X
Proximity to Major Direct Water Discharges	Toxic concentrations in stream segments within 500 meters, divided by distance in kilometers (km). Standards modeled after Risk-Screening Environmental Indicators (RSEI) [36].	X		X
Watershed Failure	Percent of each census tract’s watershed that exceeds levels of phosphorus and/or nitrogen [39].			X
Groundwater Threat	Nature and the magnitude of the threat and burden to groundwater safety posed by sites maintained in GeoTracker [35].		X	
Impaired Water Bodies	Contamination of streams, rivers, and lakes by pollutants which compromise the ability to use a body of water for drinking, swimming, fishing, aquatic life protection, etc. [35].		X	
Solid Waste Sites and Facilities	Solid waste landfills, composting, and recycling facilities [35].		X	
**Population Characteristics: Sensitive Populations**				
Asthma Emergency Discharges	Count of patients released from the hospital after being admitted for asthma or asthma-related distress [40].			X
Myocardial Infarction Discharges	Patients released from the hospital after being admitted for a heart attack or heart attack symptoms [35].		X	X
Low Birth Weight Infants	Babies born weighing less than 5.5 pounds [35].		X	X
Asthma Emergency Visits	Patients admitted to the emergency room for asthma or asthma-related distress [35].		X	
**Population Characteristics: Socioeconomic Factors**				
Percent Non-White	Percentage of individuals who define themselves as any race/ethnicity besides non-Hispanic White [35,36].	X	X	X
Percent Low-Income	Percentage of individuals whose household income in the past 12 months is less than two times below the federal poverty level [35,36].	X	X	X
Less than high school education	Percentage of individuals 25 and older who lack a high school diploma [35,36].	X	X	X
Linguistic Isolation	Percentage of households in which no one 14 years old and older speaks English "very well", or households which speak only English [35,36].	X	X	X
Individuals under age 5	Percentage of people under the age of 5 [36].	X		X
Individuals over age 64	Percentage of people over the age of 64 [36].	X		X
Unemployment	Percentage of the population over the age of 16 that is unemployed and eligible for the labor force. Excludes retirees, students, homemakers, institutionalized persons except prisoners, those not looking for work, and military personnel on active duty [35].		X	X
Housing Burdened Low Income Households	Percentage of households in a census tract that make less than 80% of the HUD Area Median Family Income and paying greater than 50% of their income to finance housing [35].		X	

**Table 2:** Additional indicators in MD EJSCREEN tool promoted by stakeholders.

Indicator	Description	Data Source and Year
Pathogenic Infrastructure	Features of the built environment that increase a population’s vulnerability to chemical and non-chemical stressors leading to adverse health outcomes. For example, liquor stores, fast food restaurants, convenience stores, pawn shops, and payday lenders [41].	American Society of Civil Engineers (ASCE), 2017
Salutogenic Infrastructure	Physical, economic, natural, social, and spiritual features of the environment that foster health and nourish wellness. For example, hospitals, primary care providers, grocery stores, parks, recreational facilities, and churches [41].	ASCE, 2017
Tree Canopy Coverage	Layer of leaves, branches, and stems of trees that cover the ground when viewed from above. Refers to the quantity and quality of trees in a specific geographic area [42].	Maryland Department of Natural Resources (DNR), 2017
Brownfields	Refer to any area that is saturated by water, either permanently or seasonally (e.g., swamps, marshes, estuaries, lakes, rivers, etc.) Can include data from monitoring sites, the location and scope of restoration efforts from Maryland’s Non-Tidal Wetland Mitigation Program and the distribution of funding provided through the Federal Clean Water Act Section 319 (h) [43].	USEPA, 2017
Wetlands and Waterways	Refer to any area that is saturated by water, either permanently or seasonally (e.g., swamps, marshes, estuaries, lakes, rivers, etc.) [44]. Can include data from monitoring sites, the location and scope of restoration efforts from Maryland’s Non-Tidal Wetland Mitigation Program and the distribution of funding provided through the Federal Clean Water Act Section 319(h) [45].	USEPA, 2017
Health & Environmental Advocacy Groups	Studies have reported a strong, inverse correlation between social capital and health inequalities [46]. Local health and environmental advocacy groups promote access to social capital and give voice to marginalized members of the community [47].	Maryland Environmental Health Network, 2017
Grocery Stores	Number and location of grocery stores including Giant, Costco, Whole Foods, Safeway, etc. [48].	North American Industry Classification System (NAICS), 2018
Convenience Stores	Number and location of convenience stores including CVS, 7-Eleven, and gas stations [49].	NAICS, 2018
Liquor Stores	Number and location of stores selling alcohol [50].	NAICS, 2018
Zoned Industrial Area—I1	Light intensity industrial zone. Manufacturing, assembling, or processing of refined goods [51].	Prince George’s (PG) County.gov
Zoned Industrial Area—I2	Heavy intensity industrial zones [51].	PG County.gov
Parks	Number and location of federal, state, and municipal parks. Includes national parks, local parks, playgrounds, biking and walking trails, etc. [52].	Protected Areas Database (PAD)-US, 2016
Recreational Areas	Green spaces used for recreation. Includes soccer fields, baseball fields, tennis courts, basketball courts, golf courses, etc. [53].	PG County Department of Parks and Recreation
Point Source Discharge	Any identifiable source from which pollutants are discharged. For example, a pipe, ditch, channel, tunnel, conduit, well, discrete fissure, container, rolling stock, concentrated animal feeding operation, or vessel or other floating craft. Agricultural stormwater discharges and return flows from irrigated agriculture are not included in this definition [54].	EPA Clean Water Act (CWA), 1972
Major Air Pollution Sources	Any source that emits 10 tons of any of the 187 toxic air pollutants listed in the Clean Air Act, or 25 tons of a mixture of air toxins, per year. There are multiple sources of pollution such as mobile (cars, trucks, and busses), stationary, (factories, refineries, and power plants), indoor (select building materials and cleaning solvents), and naturally-occurring sources (volcanic eruptions and forest fires) [55].	EPA Clean Air Act (CAA), 1992
Bladensburg Air Pollution Minor	Any source which emits, or has the potential to emit, regulated New Source Review (NSR) pollutants in amounts less than the major source thresholds [56].	CAA, 1992
Hazardous Waste Site	Any area which is contaminated by pollutants deemed dangerous or capable of having harmful effects on human health or the environment. More specifically, any area contaminated by chemicals on the F, K, P, and U lists, found in title 40, section 261, of the Code of Federal Regulations (CFR) [57].	Resource Conservation and Recovery Act (RCRA), 1976
Health Provider Shortage Area	A shortage of primary care and dental providers for the entire population or specific population groups within a defined geographic area [58].	Health Resources and Services Administration (HRSA), 2016
Supermarkets	Any establishment which retails a general line of food, such as canned, dry and frozen foods, fresh fruits and vegetables, fresh and prepared meats, fish, poultry, dairy products, baked products and snack foods. These establishments may also retail non-food products such as household paper products, toiletries, and non-prescription drugs [59].	NAICS, 2012
Limited Supermarket Access Areas	One or more contiguous census block groups where residents must travel significantly further to reach a supermarket than the, “comparative acceptable” distance that residents in well-served areas must travel [60].	The Reinvestment Fund (TRF), 2013
EPA Superfund Sites	Any land that has been identified by the USEPA as a candidate for cleanup because of hazardous waste contamination. These sites are placed on the National Priorities List (NPL) [61].	The Comprehensive Environmental Response, Compensation, and Liability Act (CERCLA), 1980
Public Schools	Public schools including elementary, middle, and high schools; charter schools; special centers [62].	Prince George’s County Public Schools (PGCPS), 2018
Railroads	Main lines such as spur lines, and rail yards, mass transit rail lines such as carlines, streetcar track, monorail or other mass transit rail and special purpose rail lines such as cog rail lines, incline rail lines, and trams [63].	US Census Bureau, Department of Commerce, 2015

**Table 3:** Comparison of usability and functionality across the screening tools.

Features	EPA EJSCREEN	CalEnviroScreen	MD EJSCREEN
Usability			
User-Defined Search	X	X	X
User-Defined Base Map Options	X		
User-Defined Location Markers	X	X	X
Zoom In/Out	X	X	X
Bookmark	X		
Help Manual	X	X	
Functionality			
Print Maps	X	X	
Share Maps		X	
Create Maps	X	X	X
Create Score		X	X
Create Reports	X		
Display Statistics		X	X
Download Raw Data	X	X	
Create Graphs	X		
Create Tables		X	X
Measure Function	X		
Locate Function	X	X	X
Display Legislative Districts			X
Display County Lines			X
Mobile Version	X		
Side-by-Side Maps	X		
Overlay Maps			X
